# Preventive Awareness and Protective Behaviours Toward Vector-Borne Diseases Among Patients at a Tertiary Care Centre in Eastern India: A Cross-Sectional Study

**DOI:** 10.7759/cureus.117636

**Published:** 2026-10-08

**Authors:** Bhagyasri Ray, Vasantha Kalyani, Tiyasha Bhowmick, Pallabi Panda, Sahina Nazmin, Abhijit Boratne

**Affiliations:** 1 College of Nursing, All India Institute of Medical Sciences, Deoghar, Deoghar, IND; 2 Department of Community and Family Medicine (CFM), All India Institute of Medical Sciences, Deoghar, Deoghar, IND

**Keywords:** attitude, chikungunya, dengue, eastern india, knowledge, lymphatic filariasis, malaria, practice, tertiary care, vector-borne diseases

## Abstract

Background: Mosquito- and arthropod-transmitted illnesses are among the leading contributors to infectious disease burden worldwide, with a disproportionate toll on tropical low- and middle-income nations such as India.The extent to which affected communities are informed about, disposed toward, and actively engaged in disease-prevention activities is widely recognised as a central determinant of control programme success.This study assessed knowledge, attitude, and practice (KAP) concerning vector-borne diseases (VBDs) among outpatient attendees at a tertiary referral facility in Eastern India and their relationships with socio-demographic characteristics.

Methods: An observational, cross-sectional survey of 546 adults (≥18 years) recruited via convenience sampling was conducted. A validated, self-administered, bilingual questionnaire assessed knowledge (15 items; score 0-15), attitude (eight Likert items; score 8-40), and practice (nine items; score 0-9). Descriptive statistics, chi-square tests, and Spearman's rank correlation were computed; significance was set at p ≤ 0.05.

Results: Most participants showed inadequate knowledge (60%; n=329), suboptimal preventive practices (84%; n=458), and a favourable attitude (58%; n=316) on the reverse-scored attitude subscale. Spearman's rank correlation confirmed significant inter-domain associations: Knowledge-Attitude (ρ = −0.625, p < 0.001), Knowledge-Practice (ρ = +0.577, p < 0.001), and Attitude-Practice (ρ = −0.752, p < 0.001). Education, occupation, marital status, religion, housing type, drainage facility, health information source, and history of VBDs were significantly associated with all three KAP domains (p < 0.05); age and income were associated with knowledge and attitude, but not practice, and gender was associated with practice only (p = 0.001).

Conclusion: Despite favourable attitudes, knowledge and practice deficits are substantial in this rural, low-income population. Targeted education delivered through healthcare workers is essential to bridge the knowledge-practice gap and reduce VBD transmission.

## Introduction

Vector-borne diseases (VBDs) account for more than 17% of all infectious diseases globally and claim in excess of 700,000 lives each year, with mosquito-transmitted diseases, including malaria, dengue, chikungunya, and lymphatic filariasis (LF), representing the greatest burden [1,2]. Globally, malaria infected an estimated 263 million people in 2023 [3]. Dengue cases rose nearly 30-fold between 2000 and 2024 to exceed 14.6 million annually [4]. Chikungunya has expanded progressively across Asia, Africa, and the Americas since 2004 [5]. LF threatens over 657 million people across 39 countries, causing chronic disability [6]. Despite decades of control efforts, VBDs persist as a major public health challenge, particularly in tropical, low-income settings where vector control infrastructure is inadequate, and community preventive behaviour remains suboptimal [7,8].

In India, VBDs impose a substantial burden on an already stretched healthcare system [9]. The country recorded approximately 255,000 malaria cases in 2024, with 83 deaths attributable to Plasmodium falciparum [10]. The National Vector Borne Disease Control Programme (NVBDCP) provides a national policy framework, yet community-level KAP gaps persist widely [10,11]. Studies across India, from urban communities in Delhi [12] and Chennai [13] to rural and slum communities in Chandigarh [14], consistently document a disconnect between disease awareness and actual preventive behaviour, shaped by educational attainment, socio-economic status, and access to health information [15,16]. Similar KAP deficits have been documented across South and Southeast Asia, including Bangladesh and Indonesia [17], underscoring that the knowledge-practice gap is a regional pattern, not an isolated local finding.

In Eastern India, the burden is amplified by concentrated rural poverty, high tribal population density, poor sanitation, and environmental conditions highly favourable to vector breeding [18]. Despite the region's high VBD endemicity, population-level data from tertiary care settings remain scarce. Adults attending tertiary outpatient departments (OPDs) represent a health-seeking, high-contact population and therefore represent an important target group for preventive education.

## Materials and methods

Study design and setting

A quantitative, cross-sectional descriptive study was conducted at the Medicine OPD of an Institute of National Importance (INI), Deoghar, India. Data collection was carried out during regular OPD hours (09:00-16:00 hours) on each working day between 1st and 28th April 2026.

Eligibility criteria

Inclusion Criteria

Participants were eligible for inclusion if they (i) were adults aged 18 years or older; (ii) were attending the Medicine OPD during the study period; (iii) were able to read and understand Hindi or English sufficiently to complete the self-administered questionnaire; (iv) had sufficient cognitive ability to understand and complete the study questionnaire.

Exclusion Criteria

Participants were excluded if they (i) were critically ill and unable to participate in the study; (ii) had already been enrolled during an earlier OPD visit in the study period; or (iii) had significant hearing, visual, or cognitive impairment that prevented questionnaire completion. 

Sample size and sampling technique

The required minimum sample was derived through application of Cochran's (1977) formula, with a reference proportion of 63.7% (p = 0.637) [19] at 95% confidence (Z = 1.96) and a 4.1% margin of error (d = 0.041), yielding a minimum of 529. After a 10% non-response adjustment, 588 questionnaires were distributed; 546 fully completed responses were analysed (response rate: 92.9%). A non-probability convenience sampling technique was used. This was chosen because the study aim was descriptive and hypothesis-generating, and daily patient attendance at a single-institution OPD is variable. It cannot be predicted in advance, so a complete sampling frame was not available. On each data-collection day, members of the research team approached eligible adult patients in the Medicine OPD waiting area after they had completed registration and before their clinical consultation so that recruitment did not interfere with clinical care. Patients were screened against the eligibility criteria, and the study purpose was explained. Those who gave informed consent received the questionnaire in their preferred language (Hindi or English). 

Data collection instrument

A self-structured, bilingual (English and Hindi), self-administered questionnaire was developed based on NCVBDC (National Center for Vector Borne Diseases Control) guidelines [20-23], with content validity confirmed by five subject experts (100% agreement post-revision). It comprised four sections: Section A: Socio-demographic profile (20 items); Section B: Knowledge (15 MCQs; scored 0-15: Good 12-15, Average 8-11, Poor 0-7); Section C: Attitude (8 Likert-scale items; scored 8-40: Favourable 8-24, Unfavourable 25-40); Section D - Practice (9 Yes/No items; scored 0-9: Good 5-9, Poor 0-4). Categories were based on cut-offs adapted from modified Bloom's criteria, expressed as a percentage of the maximum score [24,25]. For the attitude subscale, a lower composite score reflects a more favourable disposition, as items were structured such that agreement with protective statements yields lower numerical values; this reverse-scoring convention is maintained consistently in all subsequent analyses. Reliability was confirmed by pilot testing (n = 55); Cronbach's α = 0.88.

Statistical analysis

All statistical computations were performed using IBM SPSS Statistics for Windows, Version 20 (Released 2011; IBM Corp., Armonk, New York, United States); categorical variables were expressed as frequencies and percentages. Normality of the three KAP domain score distributions was assessed using the Shapiro-Wilk test, which confirmed that the data was not normally distributed (p < 0.001 for all three domains); non-parametric methods were therefore employed for all inferential analyses. Chi-square (χ²) tests assessed associations between socio-demographic variables and KAP levels. The strength of each association was measured using Cramér's V and interpreted according to Cohen's conventions, adjusted for degrees of freedom. Spearman's rank correlation (ρ) was computed for inter-domain relationships (K↔A, K↔P, A↔P), given the ordinal nature of KAP scores and non-assumption of normality. All tests were two-tailed; a p-value < 0.05 was taken as the criterion for statistical significance.

Ethical consideration

Ethical approval was secured from the Institutional Ethical Committee before data collection. The IRB approval numbers are AIIMS-Deo/RC-IEC-subcommittee/2026/84 and AIIMS-Deo/RC-IRC/March-26/633.

## Results

Socio-demographic characteristics

Among 546 participants, 34% (n=185) were aged 18-29 years, with an equal gender distribution (50% each, n=273 each). Most were married (69%, n=375), from joint families (60%, n=330), and from rural areas (72%, n=396). Primary-level education was the most prevalent (38%, n=207); 47% (n=259) were unemployed, and 48% (n=262) earned less than ₹39,830/month. The most frequently identified source of health-related information was contact with healthcare workers (40%, n=216). Comprehensive sociodemographic data are presented in Tables 1, 2.

**Table 1:** Socio-Demographic Characteristics of Study Participants: Demographic and Occupational Profile (N = 546)

Variable	Category	n	%
Age (years)	18–29	185	33.9
	30–39	146	26.7
	40–49	128	23.4
	≥50	87	15.9
Gender	Male	273	50.0
	Female	273	50.0
Marital Status	Single	136	24.9
	Married	375	68.7
	Widowed	35	6.4
Educational Status	No formal education	137	25.1
	Primary school	207	37.9
	Secondary school	116	21.2
	Graduate and above	72	13.2
	Others	14	2.6
Occupation	Unemployed	259	47.4
	Labourer	157	28.8
	Government employee	36	6.6
	Private employee	39	7.1
	Others	55	10.1
* Frequency (n) and percentage (%). Percentages may not sum to 100 due to rounding.			

**Table 2:** Socio-Demographic Characteristics of Study Participants: Residential, Economic and Environmental Profile (N = 546)

Variable	Category	n	%
Place of Residence	Rural	396	72.5
	Urban	121	22.2
	Urban slum	29	5.3
Monthly Income (INR)	Below ₹39,830	262	48.0
	₹39,830–₹59,794	140	25.6
	₹59,795–₹79,755	72	13.2
	Above ₹79,756	48	8.8
	Not disclosed	24	4.4
Type of Housing	Kutcha (temporary)	211	38.6
	Semi-pucca	190	34.8
	Pucca (permanent)	145	26.6
Type of Family	Joint	330	60.4
	Nuclear	216	39.6
Primary Info Source	Health workers	216	39.6
	Television/Radio	68	12.5
	Newspaper/Internet	92	16.8
	Friends/Family	79	14.5
	Others	91	16.7
* Frequency (n) and percentage (%). Percentages may not sum to 100 due to rounding.			

KAP score distribution

Figure 1 presents the KAP score distribution. The majority demonstrated poor knowledge (60%, n = 329) and poor preventive practice (84%, n = 458). A favourable attitude was observed in 58% of participants (n = 316). A marked attitude-practice gap was evident: positive attitudes did not translate into consistent preventive behaviour.

**Figure 1:**
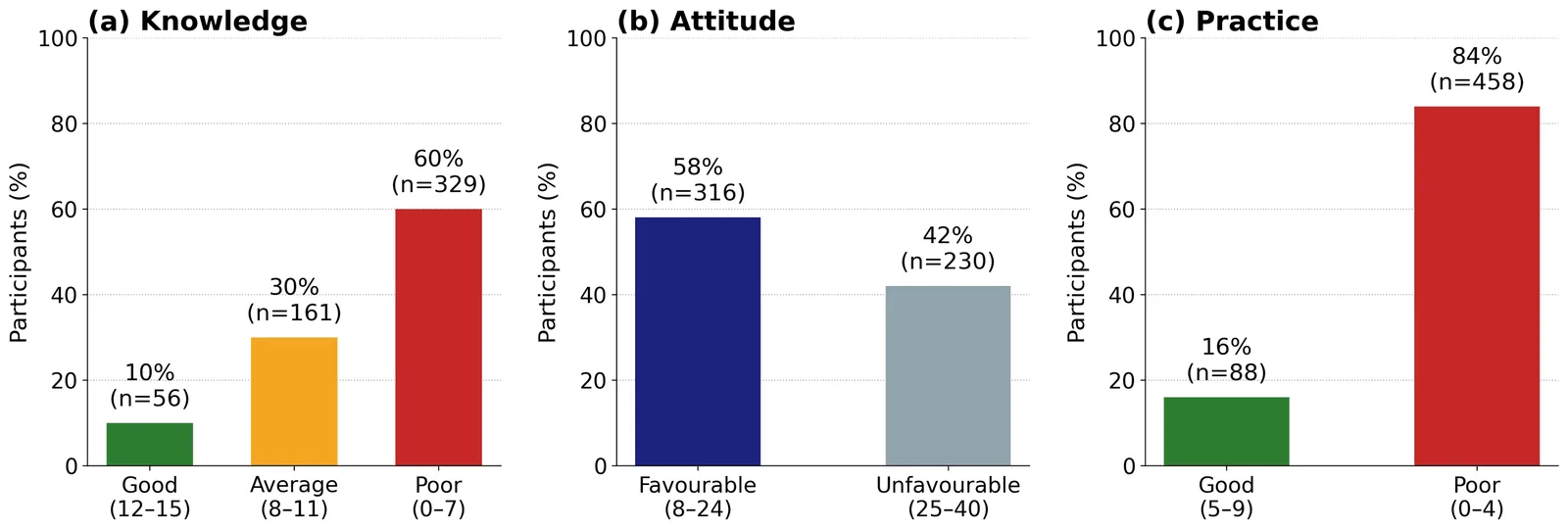
Distribution of Participants According to Knowledge, Attitude, and Practice (KAP) Levels. (a) Knowledge Levels; (b) Attitude Levels; and (c) Practice Levels.

Association of KAP with socio-demographic variables

Chi-square analyses (Table 3) identified significant associations between several socio-demographic variables and KAP levels. Educational status and source of health information were significantly associated with all three KAP domains (p < 0.001); occupation was also significant across all three domains (p < 0.001 for knowledge and attitude, p = 0.002 for practice). Age and monthly income were significantly associated with knowledge and attitude but not practice (p > 0.05). Marital status, religion, type of housing, drinking water source, and drainage facility were significantly associated with all three KAP domains (p < 0.05). Gender showed no significant association with knowledge (p = 0.069) or attitude (p = 0.666) but was significantly associated with practice (p = 0.001). The history of VBDs was significantly associated across all three domains (p < 0.05). In total, nine socio-demographic variables showed significant associations across all three KAP domains and are highlighted in bold in Table 3.

**Table 3:** Association Between Socio-Demographic Variables and KAP Domain Scores: Chi-Square Analysis (N = 546) KAP: Knowledge, attitude, and practice; VBD: vector-borne disease. Disease. χ²: chi-square test statistic. Cramer's V effect size categories: ng: negligible, sm: small, md: medium, lg: large (per Cohen's convention, adjusted for degrees of freedom). Bold rows indicate variables significant at p < .05 across all three KAP domains (Marital Status, Religion, Educational Status, Occupation, Type of Housing, Drinking Water Source, Drainage Facility, History of VBD, and Source of Health Information). † Cramer's V is reported for each KAP domain to indicate the strength of association independent of sample size. * p < .05.

Socio-demographic Variable	Knowledge		Attitude		Practice		Cramer's V†		
	χ²	p-value	χ²	p-value	χ²	p-value	Knowledge	Attitude	Practice
Age	29.905	<0.001*	32.529	0.001*	2.101	0.552	0.165 (sm)	0.244 (sm)	0.062 (ng)
Gender	5.345	0.069	0.22	0.666	10.454	0.001*	0.099 (ng)	0.020 (ng)	0.138 (sm)
Marital Status	36.959	<0.001*	26.089	<0.001*	11.254	0.004*	0.184 (sm)	0.219 (sm)	0.144 (sm)
Religion	20.874	0.002*	45.004	<0.001*	17.828	<0.001*	0.138 (sm)	0.287 (sm)	0.181 (sm)
Educational Status	47.986	<0.001*	101.201	<0.001*	40.655	<0.001*	0.210 (sm)	0.431 (md)	0.273 (sm)
Occupation	51.473	<0.001*	51.892	<0.001*	17.215	0.002*	0.217 (md)	0.308 (md)	0.178 (sm)
Type of Family	0.492	0.782	50.724	<0.001*	25.585	<0.001*	0.030 (ng)	0.305 (md)	0.216 (sm)
Place of Residence	5.189	0.268	12.344	0.002*	3.169	0.205	0.069 (ng)	0.150 (sm)	0.076 (ng)
Duration of Stay	6.991	0.136	15.805	<0.001*	26.006	<0.001*	0.080 (sm)	0.170 (sm)	0.218 (sm)
Monthly Income	35.7	<0.001*	83.318	<0.001*	5.976	0.309	0.181 (sm)	0.391 (md)	0.105 (sm)
Type of Housing	22.392	<0.001*	59.288	<0.001*	14.055	0.001*	0.143 (sm)	0.330 (md)	0.160 (sm)
Drinking Water Source	15.012	0.020*	30.51	<0.001*	20.302	<0.001*	0.117 (sm)	0.236 (sm)	0.193 (sm)
Drainage Facility	33.706	<0.001*	50.368	<0.001*	10.253	0.006*	0.176 (sm)	0.304 (md)	0.137 (sm)
Stagnant Water Near Residence	7.206	0.027*	9.613	0.002*	0.425	0.516	0.115 (sm)	0.133 (sm)	0.028 (ng)
Domestic Animals Near Residence	7.965	0.019*	0.464	0.278	5.72	0.022*	0.121 (sm)	0.029 (ng)	0.102 (sm)
Sanitation Facility	2.54	0.281	18.218	<0.001*	13.14	<0.001*	0.068 (ng)	0.183 (sm)	0.155 (sm)
History of VBD	9.569	0.008*	4.524	0.038*	8.557	0.003*	0.132 (sm)	0.091 (ng)	0.125 (sm)
Source of Health Information	41.581	<0.001*	130.012	<0.001*	42.834	<0.001*	0.195 (sm)	0.488 (md)	0.280 (sm)

Spearman's rank correlation between KAP domain scores

All three inter-domain correlations were statistically significant (p < 0.001), as shown in Table 4. The knowledge-attitude correlation (ρ = −0.625) confirmed that higher knowledge corresponded with more favourable attitudes (the negative sign reflects the reverse-scored attitude subscale; a higher knowledge score corresponds to a lower, more favourable attitude score). The knowledge-practice correlation (ρ = +0.577) indicated that better knowledge was associated with improved preventive practices. The strongest association was observed between attitude and practice (ρ = −0.752), indicating the strongest association between attitude and preventive behaviour.

**Table 4:** Spearman's Rank Correlation Between Knowledge, Attitude, and Practice Domain Scores

Domain	Knowledge (K)	Attitude (A)	Practice (P)
Knowledge (K)	1.000	−0.625***	+0.577***
Attitude (A)	−0.625***	1.000	−0.752***
Practice (P)	+0.577***	−0.752***	1.000
§ *** p < 0.001 (two-tailed); Spearman's ρ; N = 546. Diagonal values = 1.000 (self-correlation).			

## Discussion

This study shows VBD-related preventive behaviour among 546 adults at a tertiary care setting in Eastern India. Seventy-two percent of the sample lived in rural areas, 48% had low incomes, and their educational attainment was low to moderate.

Knowledge

The results of the present study show that poor knowledge was found in 60% of participants, consistent with global and Indian evidence. Poor knowledge, ranging from 53%, was reported in rural Uttar Pradesh, with education and age identified as the strongest predictors [15]. Similarly, low community knowledge of dengue and chikungunya has been documented in Tanzania, where only 15% of community members achieved good dengue knowledge scores [26]. A community-based study in Ethiopia found that only 45% of participants knew that a virus caused chikungunya, and only 16% correctly identified the Aedes' daytime-biting pattern [27]. In French Guiana, chikungunya was found to be the least well-understood mosquito-borne disease among high school students, despite its perceived severity [28].

In contrast, studies conducted in urban areas with better access to mass media and formal education report higher knowledge levels. Awareness of mosquito-borne diseases as high as 92% has been reported from a peri-urban village in Delhi [12], while 89% of urban participants in Telangana could name at least one mosquito-borne disease [16]. Near-universal knowledge 95% regarding mosquito-borne transmission was documented in urban Karnataka [29]. Excellent knowledge of the mosquito-malaria link has also been reported among urban residents in Mumbai. However, persistent misconceptions about transmission routes were noted [30]. The lower knowledge scores observed in the present study reflect the predominantly rural, low-education context of Eastern India, where health literacy and media penetration remain limited.

Attitude

In this study, a favourable attitude was observed in 57% of participants, which was higher than the 32% reported in Indonesia [17] and the 46% reported in rural Ethiopia [19]. Educated, higher-income, and nuclear-family participants were significantly more likely to hold favourable attitudes, consistent with evidence from urban Nepal (91% positive attitudes) [31]. Filariasis-related stigma was observed among less-educated and rural participants, as similarly documented in Nigeria [32], underscoring the need for destigmatisation alongside clinical education.

Practice

The most concerning finding was poor preventive practice in 84% of participants, despite a majority holding favourable attitudes. This knowledge-practice gap has been consistently documented across settings. Good malaria-prevention practices as low as 40% and bed net usage of only 10% have been reported in Ghana [33], while bed net use of just 30% was observed in Mumbai despite adequate knowledge levels [30]. In Mauritius, repellent cream use was reported by only 20% of participants, and protective clothing by 34% [34].

From an Indian perspective, a more diverse range of protective measures has been documented in metropolitan Chennai, where 40% of participants used liquidators, 31% used mosquito coils, and 9% used repellent creams, reflecting how urbanisation and higher awareness can translate into broader practice adoption [13]. Persistently low vector-control practices have also been reported in urban slums of Eastern India, even among disease-aware populations [18], suggesting that structural barriers compound knowledge and attitudinal gaps in this region.

For lymphatic filariasis specifically, fewer than half of mass drug administration (MDA) drug deliverers in Indonesia were found to have adequate filariasis knowledge, directly limiting community MDA uptake [35]. These were consistent with the present finding that only 47% of participants reported taking MDA tablets during the last distribution round. Poor mosquito net use of 61% in rural low-resource settings further reinforces the practice gap [32].

Spearman's rank correlation findings

The strongest correlation was observed between attitude and practice (ρ = −0.752, p < 0.001). The Knowledge-Attitude (ρ = −0.625) and Knowledge-Practice (ρ = +0.577) correlations were also significant (p < 0.001), consistent with knowledge and attitude both being associated with behaviour. The stronger attitude-practice association suggests that attitude-change interventions may be more effective than knowledge-only education. These findings are consistent with KAP inter-domain correlations reported in Indonesia [17] and Mauritius [34]. 

Attitude-change interventions may yield more substantial behavioural returns than programmes focused exclusively on knowledge transfer. These inter-domain patterns are concordant with those documented in Indonesian and Mauritian KAP investigations [17,34].

Limitations

The cross-sectional design precludes causal inference, and convenience sampling at a single health facility limits generalisability to other settings, populations, and regions. Future studies should employ multicentric, probability-based sampling and longitudinal or interventional designs to evaluate whether KAP improvements translate into measurable reductions in VBD incidence. Some knowledge items used the scientific names of vectors, which may have been unfamiliar to participants with low literacy and could have led to an underestimation of knowledge scores.

## Conclusions

This study's findings show significant deficits in VBD preventive awareness and behaviour among adults in Eastern India, with poor knowledge and poor preventive practice despite favourable attitudes in the majority. A consistent association across knowledge, attitude, and practice domains was observed, with attitude showing the strongest correlation with behaviour. Socio-economic disadvantage and low health literacy are key drivers. Nurse-led community education, strengthened NVBDCP outreach, and future interventional studies are suggested to bridge this gap.

## Appendices

Annexure I 

Data Collection Tool - English Version

Section A: Sociodemographic Profile

(To be filled by interviewer/respondent)

1. Age (in completed years): _______

a) 18-29 b) 30-39 c) 40-49  d) >50

2. Sex:

a) Male b) Female c) Other

3. Marital status: a) Single b) Married c) Widowed d) Divorced / Separated

4. Religion:

a) Hindu  b) Muslim c) Christian  d) Others (specify): _________ 5. Education:

a) Illiterate b) Primary school c) Middle school d) Secondary / Higher secondary e) Graduate and above

6. Occupation:

a) Unemployed b) Student c) Housewife d) Farmer e) Laborer     f) Service / Private job g) Government employee h) Business / Self-employed

7. Type of family:

a) Nuclear family Joint family

8. Family size:

a) ≤4 members b) 5-7 members c) >7 members

9. Family monthly income (₹):

a) <₹10,000 b) ₹10,001-20,000 c) ₹20,001-30,000 d) ₹30,001-50,000 e) >₹50,000

10. Place of residence:

a) Urban  b) Rural c) Urban slum

11. Duration of stay at current residence:

a) <1 year b) 1-5 years c) >5 years

12. Socio-economic status (Modified BG Prasad scale):

a) Upper class (>₹159,587)

b) Upper middle class (₹79,756-₹159,585)

c) Lower middle class (₹59,759-₹79,755)

d) Upper lower class (₹39,830-₹59,794)

e) Lower class (<₹39,830)

f) Others

13. Type of housing:

a) Kutcha b) Semi-pucca c) Pucca

14. Availability of drinking water:

a) Piped water b) Hand pump / Tube well c) Well d) Other (specify): __________

15. Drainage facility around house:

a) Open drainage b) Closed drainage c) No drainage

16. Presence of stagnant water near residence:

a) Yes b) No

17. Presence of domestic animals near residence:

a) Yes b) No

If Yes, specify: …………………………

18. Sanitation facility at home:

a) Toilet present b) No toilet

19. History of vector-borne disease in the past (self or family):

a) Yes b) No

If yes, specify disease: ☐ Malaria  ☐ Dengue  ☐ Chikungunya  ☐ Filariasis

20. Source of health information regarding vector-borne diseases:

a) Health workers b) Television / Radio c) Newspaper / Internet d) Friends / Family     e) Others (specify): __________

Section B: Knowledge

1. Which mosquito transmits malaria?

a) Anopheles b) Aedes c) Culex     d) Psorophora

2. Common early symptoms of malaria include:

a) Fever b) Cough c) Skin rash d) Others

3. How soon should someone with suspected malaria seek medical care?

a) Within 24 hours b) After 3 days c) After a week d) After a month

4. Which one helps to prevent malaria?

a) Eating high-protein food b) Using insecticide-treated mosquito nets c) Drinking warm water d) Wearing bright-coloured clothes

5. Which mosquito species mainly transmits dengue?

a) Aedes aegypti     b) Anopheles c) Culex     d) Psorophora

6. When does the Aedes mosquito bite most often?

a) Daytime b) Nighttime c) Evening time d) Both daytime & nighttime

7. Common symptoms of dengue include:

a) Fever b) Joint pain c) Rash d) All of the above

8. Which of the following is an important breeding site for Aedes?

a) Discarded containers with clean water b) Flowing river c) Dry soil d) Open field

9. Chikungunya is transmitted by which mosquito?

a) Aedes b) Anopheles c) Culex d) Psorophora

10. A key symptom of chikungunya is:

a) Severe joint pain b) Itchy eyes c) Jaundice d) Others

11. What are the preventive actions for chikungunya?

a) Wearing thick winter clothes only b) Avoiding drinking water during daytime c) Preventing mosquito bites by using repellents and eliminating stagnant water d) Eating spicy food to increase immunity

12. Lymphatic filariasis is caused by tiny worms transmitted by which vector?

a) Culex b) Anopheles c) Aedes d) Psorophora

13. What are the common chronic signs of filariasis?

a) High fever with chills only b) Hydrocele, elephantiasis, and lymphoedema c) Sudden paralysis of limbs d) Skin rashes that disappear within hours

14. In which part of the human body do filarial worms mainly reside and cause damage?

a) Respiratory tract b) Lymphatic system c) Digestive system d) Nervous system

15. Which of the following is an effective preventive strategy for lymphatic filariasis at the community level?

a) Regular mass drug administration with DEC (diethylcarbamazine) b) Increasing intake of vitamin supplements c) Avoiding cold drinks during the monsoon d) Wearing only light-coloured clothing

Section C: Attitude

**Table 5:** Attitude scale (Strongly Agree = 1 to Strongly Disagree = 5)

S.No.	Item	Strongly Agree (1)	Agree (2)	Neutral (3)	Disagree (4)	Strongly Disagree (5)
1	Malaria is a serious health threat in my community.					
2	I trust that insecticide-treated nets are effective to prevent malaria.					
3	Dengue is a serious and preventable disease in my area.					
4	I believe community clean-up campaigns reduce dengue risk.					
5	Chikungunya can cause long-term health problems and should be taken seriously.					
6	If someone has joint pain and fever, they should see a health facility promptly.					
7	People with swollen limbs due to filariasis should not be stigmatized.					
8	I believe community participation is essential to elimination of filariasis.					

Section D: Practice

1. Do you sleep under an insecticide-treated bed net regularly?

a) Yes b) No

2. Do you close doors/windows or use screens at night to avoid mosquitoes?

a) Yes b) No

3. Do you participate in community IRS (Indoor Residual Spraying) or accept house spraying if offered every 6 months?

a) Yes b) No

4. Do you use any preventive measures during daytime in mosquito-prone areas?

a) Yes b) No

5. Have you participated in any community vector-control / cleanup activity in the last 12 months?

a) Yes b) No

6. If you have a fever with a body ache, do you seek medical testing (blood test) at a facility?

a) Yes b) No

7. Do you regularly remove standing water or cover containers to prevent mosquito breeding?

a) Yes b) No

8. In the last MDA (Mass Drug Administration) round, did you take the distributed tablets?

a) Yes b) No

9. Do you avoid open water or mosquito breeding sites around the house to reduce filariasis risk?

a) Yes b) No

Answer Key - Section B (Knowledge)

1. A

2. A

3. A

4. B

5. A

6. A

7. B

8. A

9. A

10. A

11. C

12. A

13. B

14. B

15. A
